# The value of correlation of serum 20S proteasome concentration and percentage of lymphocytic apoptosis in critically ill patients: a prospective observational study

**DOI:** 10.1186/cc9340

**Published:** 2010-11-25

**Authors:** Ayman A Yousef, Ghada A Suliman, Maaly M Mabrouk

**Affiliations:** 1 Department of Anesthesia, Tanta University Hospitals, El-Geish Street, Tanta 31527, Egypt; 2 Department of Clinical Pathology, Tanta University Hospitals, El-Geish Street, Tanta 31527, Egypt

## Abstract

**Introduction:**

Sepsis in critically ill patients is almost associated with bad prognosis and its early detection may improve the prognosis. However, it is difficult to monitor the immunological state of these patients depending on the traditional markers of infection or inflammatory mediators. Accelerated lymphocyte death may reflect good idea about the prognosis especially when combined with 20S proteasome determinations, a recently discovered marker for muscle degradation in patients with sepsis. The hypothesis of the present study is to evaluate the role of serum 20S proteasome at early diagnosis of sepsis and its correlation with lymphocyte apoptosis to predict prognosis and consequently the early interference in critically ill patients suffering from a broad range of diseases in the intensive care unit.

**Methods:**

Sixty-seven critically ill adult intensive care patients were divided into two groups, 32 septic critically ill patients (sepsis group) and 35 non-septic critically ill patients (non-sepsis group), in addition to 33 apparently healthy subjects from the out patient clinic (control group). Patients were tested for serum values of 20S proteasome using ELISA and for percentage of lymphocyte death using annexin V and 7-aminoactinomycin D dye by flow cytometry.

**Results:**

Measured median value of serum 20S proteasome was significantly higher in septic patients compared with both the non-septic and control groups. A significant increase in the percentage of apoptotic lymphocytes was detected in septic patients when compared with the non-sepsis and control groups. The correlation of both 20S proteasome and percentage of apoptotic lymphocytes was found to be significantly positive in both septic and non-septic patients.

**Conclusions:**

The correlation of median values of 20S proteasome and the percentage of apoptotic lymphocyte median values could be a good indicator of patient prognosis and survival in critically ill patients.

## Introduction

Sepsis in critically ill patients correlates with bad prognosis. Previous studies have tried to monitor biomarkers for the detection of sepsis, however none of these markers provided a good idea about the immunologic state of the patients, thus, the need for immunologic markers for early detection of an immunocompromised state in critically ill patients is essential [1].

Increased lymphocyte apoptosis is one of the suggested causes of immunosuppression in critically ill patients. In the presence of a hyperinflammatory state, apoptosis may be beneficial to the host by eliminating lymphocytes that produce excessive pro-inflammatory cytokines. Conversely, lymphocyte apoptosis could be harmful in sepsis by causing depletion of lymphocytes that are essential for defense against invading microorganisms [2].

Muscle cachexia and degradation of myofibrillar proteins is another common important clinical feature in critically ill septic patients [3]. Recently, a study in patients with sepsis confirmed that muscle catabolism in patients with sepsis is associated with upregulated energy-ubiquitin-dependent protein breakdown [4]. In this proteolytic pathway, proteins are conjugated to ubiquitin, then they are degraded by the 26S proteolytic complex [5], which is composed of a core known as 20S proteasome which is composed of seven different α and β subunits arranged in four heptameric rings [6]. Increased level of circulating 20S proteasome was proposed as a marker of cell damage and protein breakdown in critically ill septic patients. The study hypothesis is to determine the role of serum 20S proteasome at early diagnosis of sepsis and its correlation with lymphocyte apoptosis to predict prognosis and early interference in critically ill patients suffering from a broad range of diseases in the intensive care unit (ICU).

## Materials and methods

After the study approval by an Investigational Review Board of the Faculty of Medicine, Tanta University, an informed consent was obtained from all patients who were able to grant such consent prospectively; otherwise, consent was obtained retrospectively or from the patient's next-of-kin. The study was conducted over one year in the ICU of the Emergency Hospital of Tanta University, Tanta, Egypt. It is 25-bed medical/surgical ICU.

Sixty-seven critically ill adult intensive care patients divided into two groups, 32 septic patients (sepsis group) and 35 nonseptic critically ill patients (nonsepsis group), and 33 apparently healthy subjects from the outpatient clinic (control group) were observed regarding 20S proteasome and the percentage of lymphocyte apoptosis. Patients who received corticosteroids or anti-inflammatory drugs before admission, who had immunosuppressive illness, who had chronic organ failure, who received massive blood transfusion, who received radiation therapy or who had previous organ transplantation were excluded from the study. At admission, the patient's age, sex, height and weight were measured. Patients' data include clinical status, Sequential Organ Failure Assessment (SOFA) score, blood pressure, heart rate, respiratory rate, temperature, central venous pressure, laboratory analysis (complete blood count, serum sodium, potassium, calcium, blood urea nitrogen, blood sugar, prothrombin time, aspartate aminotransferase, alanine aminotransferase, albumin and C-reactive protein, and arterial blood gas analysis were recorded. Routine cultures of urine, blood and suspected areas were obtained to determine the presence of infection. We attempted to maintain the patient central venous pressure at 8 to 12 cmH_2_O and the hemoglobin level at 10 to 12 g/dl. Whenever needed, intravascular fluid replacement, blood products, vasopressor and/or inotropic agents were administered. The physician in the ICU evaluated all of the study patients daily for sepsis, severe sepsis, or septic shock.

The signs of sepsis were body temperature <33.6°C or >38.3°C, tachycardia (>90 beats/minute), ventilatory frequency >20 breath/minute or pressure of carbon dioxide <32 mmHg (unless the patient was mechanically ventilated), a white cell count ≥12 × 10^9^/l or <4 × 10^9^/l, or >10% immature neutrophils, in addition to the presence of infection [7]. Severe sepsis was considered as sepsis with evidence of organ dysfunction and hypoperfusion, acute alteration of mental status, elevated plasma lactate, unexplained metabolic acidosis (arterial pH <7.3), hypoxemia, a decrease in platelet count >50% or ≤100 × 10^9^l/l or prolonged prothrombin time, oliguria and hypotension defined as systolic arterial pressure <90 mmHg or a decrease >40 mmHg. Septic shock was considered as hypotension (<90/60 mmHg) in addition to sepsis syndrome persisting despite adequate fluid resuscitation and requiring intropic support. SOFA score consists of scores from six organ systems (respiratory, cardiovascular, hepatic, coagulation, renal, and neurological) graded from 0 to 4 according to the degree of dysfunction/failure. The aggregate score (total maximum SOFA score) is calculated summing the worst scores for each of the organ systems during the ICU stay [8].

### Estimation of 20S proteasome

Microtitration plates coated with monoclonal antibody to 20S proteasome were used. Human sera samples diluted 1:20 were applied to each well for 3 hours at room temperature. A standard curve was established using 20S proteasome standard preparation having concentrations of 5,000 ng/ml to 78 ng/ml (six linear dilution steps). After a washing step, a polyclonal antibody to 20S proteasome α and β subunits was added for 2 hours, followed by another washing step. Peroxidase-conjugated anti-rabbit IgG was used for detection of the antigen, incubated for 1 hour, substrate was added (tetramethyl benzidine) and finally the reaction was stopped with sulfuric acid. Optical density values were determined at 450 nm.

A negative control in the form of bovine serum albumin was used to exclude nonspecific reaction to proteins and no reaction was detected. A positive control in the form of human placental proteasome preparation was also used (AFFINTI Research Products Ltd, Mam Head, Exeter, UK).

### Estimation of the percentage of apoptotic lymphocytes by flow cytometry

Whole blood on ethylenediamine tetraacetic acid vacutainer samples were used. Red blood cells were lysed with ammonium chloride 1.0 mM and white blood cells were washed three times with PBS. Cells were incubated for 30 minutes in the dark with the monoclonal antibody for the target CD cells or with the dyes used (annexin V and 7-aminoactinomycin D (7-AAD); Becton Dickinson and Pharmingen (St. Jose, California, USA)). Forward and side scatter properties for lymphocytes were used with the use of a pan-lymphocyte, B-lymphocyte and T-lymphocyte panel including CD19 labeled with cy 5 dye for B cells and CD3 labeled with PerCP dye for T cells. With the use of annexin V labeled with fluorescein isothiocyanate (FITC) and 7-AAD labeled with phycoerythrin staining for apoptotic lymphocytes, the percentage of these cells was detected. Detection of apoptosis using annexin V was accompanied with the use of 7-AAD detection kits. The final combination used for all patients was (CD19/annexin V/CD3/7-AAD).

FITC annexin V staining precedes the loss of membrane integrity that accompanies the latest stages of cell death either due to apoptotic or necrotic processes. Staining with FITC annexin V is therefore typically used with a vital dye such as 7-AAD to identify the early apoptotic cells (phycoerythrin 7-AAD-negative/FITC annexin V-positive) and to differentiate the late apoptotic or dead cells (positive for both 7-AAD and annexin V) from viable cells that are negative for both 7-AAD and annexin V [9-11].

## Results

A total of 100 patients (59 men and 41 women) were included in the study. Thirty-two patients developed septic complications during the ICU stay (sepsis group), 10 patients developed septic shock, 15 patients developed severe sepsis, and 7 patients developed sepsis without any organ dysfunction. Thirty-five patients were critically ill without evidence of infectious organism (non-sepsis group), 10 patients developing non-septic complications in the form of disturbed hepatic or renal functions, electrolyte imbalance or acid-base disorders, in addition to thirty three non-critically ill non-septic patients (control group). No significant difference was detected among the groups except for SOFA score at ICU admission and the duration of the stay in the ICU, which were higher in septic patients (Table 1).

**Table 1:** Patient characteristics

	Sepsis group (*n *= 32)	Non-sepsis group (*n *= 35)	Control group (*n *= 33)
Age (years)	44 ± 9.5	45 ± 8.7	44 ± 10.2
Sex ratio (male/female)	19/13	21/14	19/14
SOFA score	12 (7-14)*	**6 (3-10)**	
Duration of ICU stay (days)	16.9 ± 4.6*	5.8 ± 2.7	
Diagnosis			
Respiratory insufficiency due to:			
Bacterial infection	6		
ARDS	4		
COPD		2	
Bronchial asthma		4	
Pulmonary edema		3	
Polytrauma	7	8	
Orthopedic surgery	9	11	
Thoracic surgery	6	7	

Data presented as mean and standard deviation or (interquartile range for SOFA). *Significant change, *P *< 0.05. ARDS, adult respiratory distress syndrome; COPD, chronic obstructive pulmonary disease; ICU, intensive care unit; SOFA, Sequential Organ Failure Assessment.

**Figure 1:**
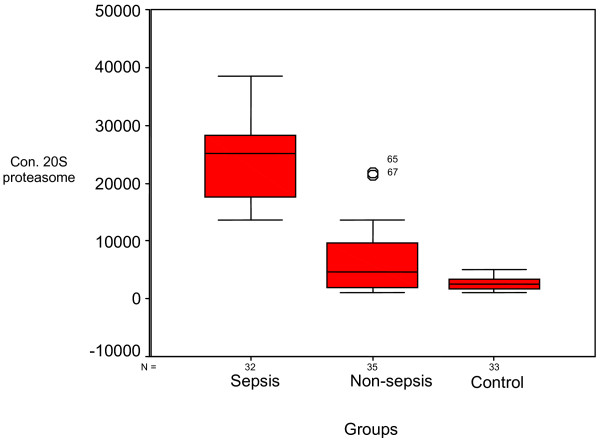
**Comparison between concentrations of 20 S proteasome in the studied groups**.

There was a significant variation among median values of 20S proteasome in the studied groups: a median value of 25,125 ng/ml in the sepsis group, a median value of 4,560 ng/ml in the nonsepsis group, and a median value of 2,740 ng/ml in the control group. The mean rank was 83.69, 42.66 and 26.64 for the studied group, respectively. The sepsis group showed the highest values, followed by the nonsepsis group and lastly the control group (*P *< 0.001) (Table 2 and Figure 1).

**Table 2:** Comparison of the concentration of 20 S proteasome in the studied groups

	20 S proteasome		
	Range	Median	Mean rank
Sepsis group	13,700 to 38450	25,125.00	83.69
Non-sepsis group	1,170 to 21710	4,590.00	42.66
Control group	1,130 to 4970	2,470.00	26.64
			
Kruskal-Wallis test	χ^2 ^= 66.764	*P *= 0.000*	
			
Mann-Whitney test	Sepsis and nonsepsis groups	Sepsis and control groups	Nonsepsis and control groups
*P *value	<0.001*	<0.001*	0.001*

*Significant change, *P *< 0.05.

**Figure 2:**
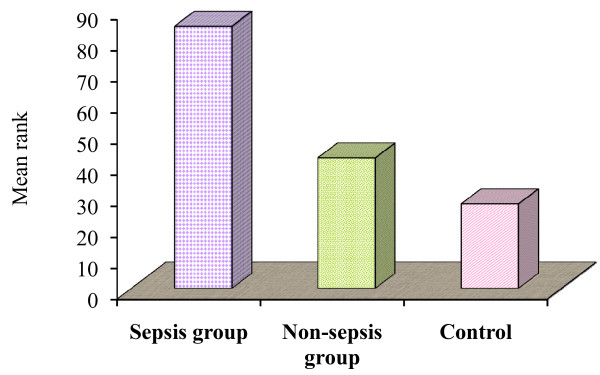
**Comparison of percentage of apoptotic lymphocytes in the studied groups**.

There was a significant variation of the median values among the studied groups regarding the percentage of apoptotic lymphocytes: the median value for the sepsis group was 11.75%, the median value for the non-sepsis group was 3.6%, while the median value for the control group was 2.2%. The median rank for the studied groups was 84.34, 41.9 and 26.8, respectively. The sepsis group showed the highest values, followed by the non-sepsis group and lastly the control group (Table 3 and Figure 2).

**Table 3:** Percentage of total apoptotic lymphocytes among the studied groups

	Range	Median	Mean rank
Sepsis group	8.200 to 18.400	11.750	84.344
Non-sepsis group	1.500 to 9.600	3.600	41.971
Control group	1.300 to 6.400	2.200	27.176
			
Kruskal-Wallis test	χ^2 ^= 68.506	*P *= 0.000*	
			
Mann-Whitney test	Sepsis and non-sepsis groups	Sepsis and control groups	Non-sepsis and control groups
*Z*	-6.917	-6.982	-3.057
*P *value	0.000*	0.000*	0.002*

*Significant change, *P *< 0.05.

**Figure 3:**
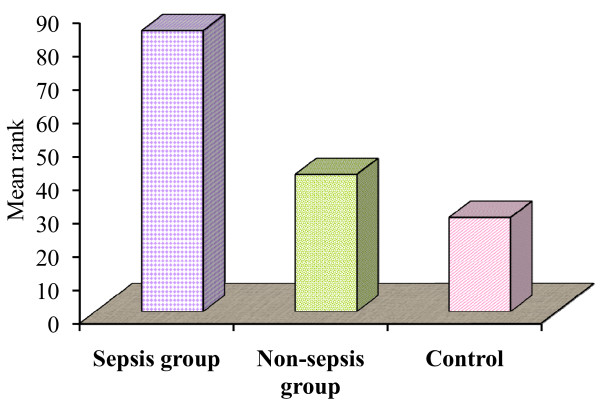
**Percentage of apoptotic B lymphocytes among the studied groups**.

**Figure 4:**
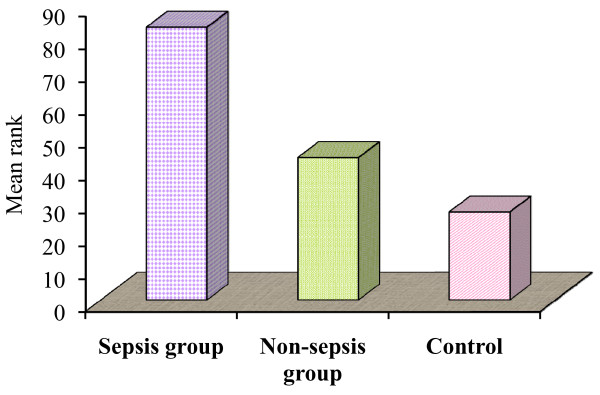
**Percentage of apoptotic T lymphocytes among the studied groups**.

A significant variation of the percentage of apoptotic B lymphocytes was observed in the studied groups: in the sepsis group the median value was 5.9% and the median rank 84.15, while this was 1.8% with a median rank of 41.1 in the non-septic group, and the median value in the control group was 1.09% with a median rank of 28.2 (*P *= 0.008). Similarly, a significant variation of the percentage of apoptotic T lymphocytes was observed in the studied groups: in the sepsis group the median value was 5.9% and the median rank 83.1, while this was 1.7% with a median rank of 43.4 in the non-septic group, and the median value in the control group was 2.2% with a median rank of 27.1 (*P *= 0.001) (Tables 4 and 5, and Figures 3 and 4).

**Table 4:** Percentage of apoptotic B lymphocytes among the studied groups

	Range	Median	Mean rank
Sepsis group	3.762 to 9.384	5.942	84.156
Non-sepsis group	0.525 to 4.800	1.836	41.118
Control group	0.663 to 4.425	1.097	28.206
			
Kruskal-Wallis test	χ^2 ^= 66.721	*P *= 0.000*	
			
Mann-Whitney test	Sepsis and non-sepsis groups	Sepsis and control groups	Non-sepsis and control groups
*Z*	-6.878	-6.942	-2.663
*P *value	0.000*	0.000*	0.008*

*Significant change, *P *< 0.05.

**Table 5:** Percentage of apoptotic T lymphocytes among the studied groups

	Range	Median	Mean rank
Sepsis group	2.375 to 9.016	5.914	83.156
Non-sepsis group	0.735 to 5.785	1.738	43.412
Control group	0.637 to 3.190	1.054	26.853
			
Kruskal-Wallis test	χ^2 ^= 65.184	*P *= 0.000*	
			
Mann-Whitney test	Sepsis and non-sepsis groups	Sepsis and control groups	Non-sepsis and control groups
*Z*	-6.480	-6.929	-3.240
*P *value	0.000*	0.000*	0.001*

*Significant change, *P *< 0.05.

**Figure 5:**
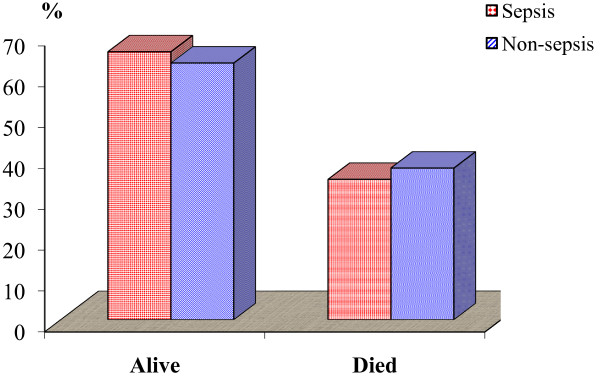
**Survival rate between the sepsis and non-sepsis groups**.

Concerning the survival rate for the sepsis and nonsepsis groups, in the sepsis group 21 patients recovered and 11 patients died during the ICU stay compared with 22 patients recovering and 13 patients dying during the ICU stay in the nonsepsis group. There was no significant variation between the two groups concerning the survival rate (Table 6 and Figure 5).

**Table 6:** Survival rates among the sepsis and non-sepsis groups

		Survival		
		Alive	Died	Total
Sepsis group	*n*	21	11	32
	%	65.63	34.38	100.00
Non-sepsis group	*n*	22	13	35
	%	62.86	37.14	100.00
Total	*n*	43	24	67
	%	64.18	35.82	100.00
Chi-square test	χ^2^	0.056		
	*P *value	0.813		

**Figure 6:**
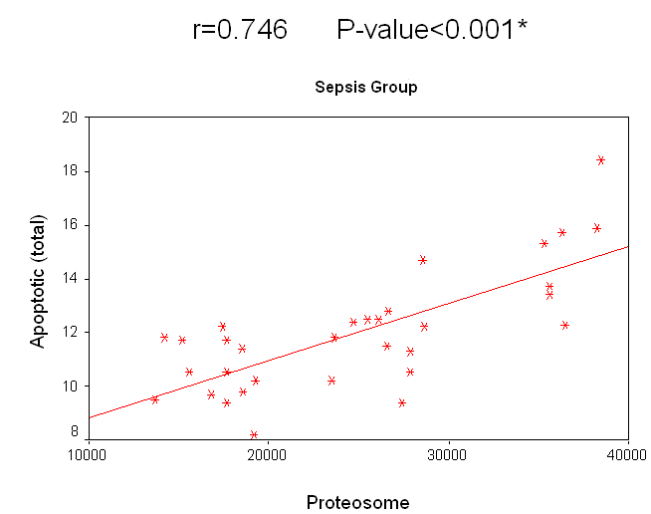
**Correlation between 20 S proteasome and the percentage of apoptotic total lymphocytes in the sepsis group**.

**Figure 7:**
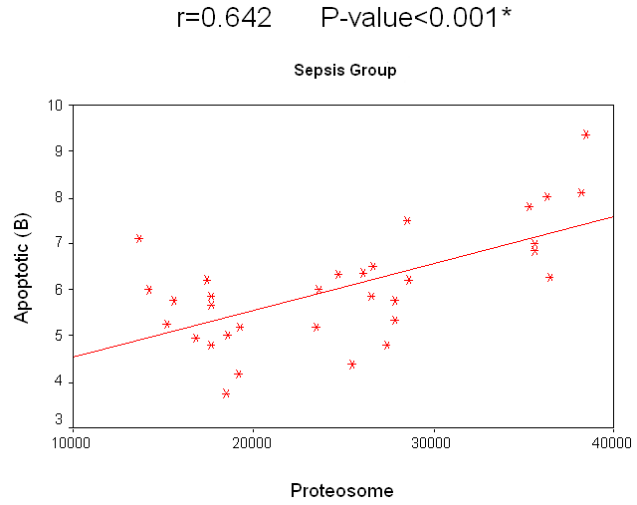
**Correlation between 20 S proteasome and the percentage of apoptotic B lymphocytes in the sepsis group**.

**Figure 8:**
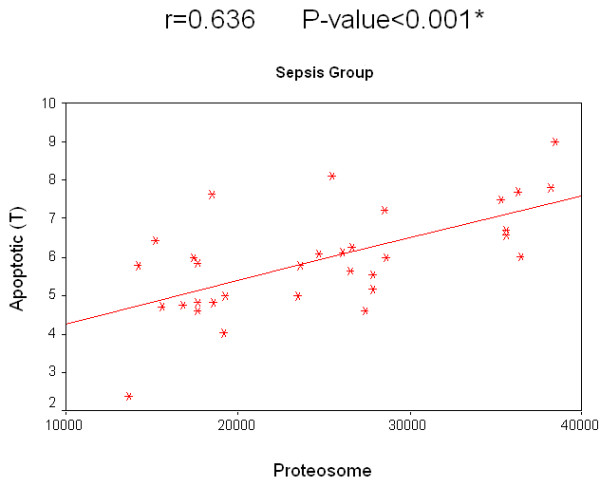
**Correlation between 20 S proteasome and the percentage of apoptotic T lymphocytes in the sepsis group**.

**Figure 9:**
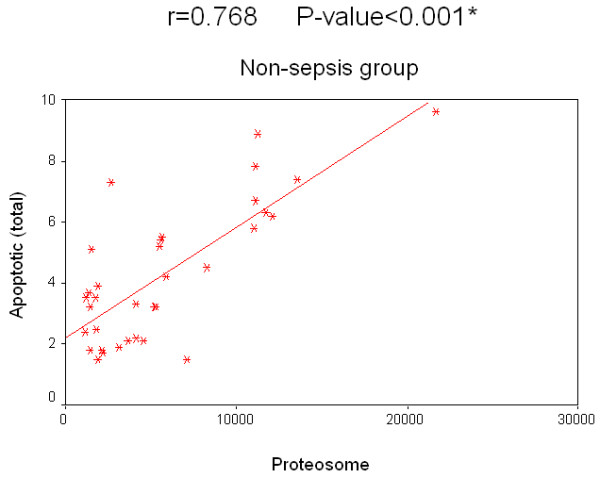
**Correlation between 20 S proteasome and the percentage of apoptotic total lymphocytes in the non-sepsis group**.

**Figure 10:**
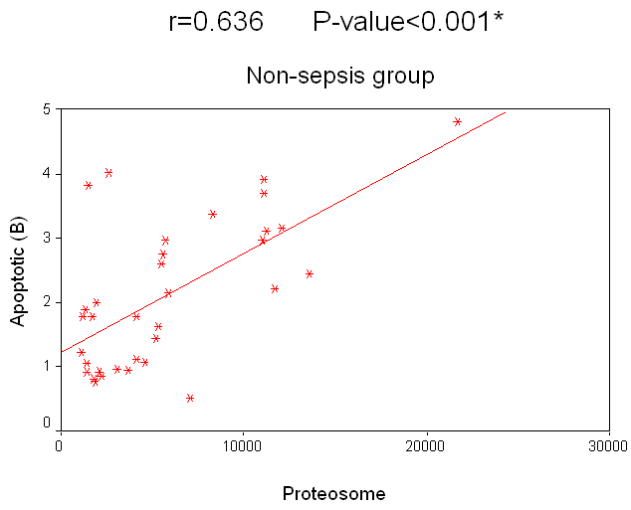
**Correlation between 20 S proteasome and the percentage of apoptotic B lymphocytes in the non-sepsis group**.

**Figure 11:**
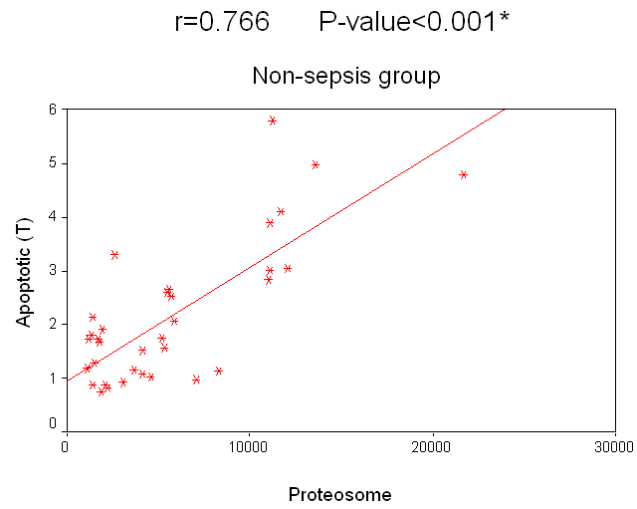
**Correlation between 20 S proteasome and the percentage of apoptotic T lymphocytes in the non-sepsis group**.

Concerning the relation of survival to the concentration of 20S proteasome in the sepsis group, survived patients had a median value of 26,150 ng/ml compared with 19,200 ng/ml in patients who did not survive. The relation of survival to the percentage of apoptotic lymphocytes in the sepsis group, the median value of survived patients was 12.2% compared to 10.5% in non-survived patients. Regarding non-sepsis group, the median value for serum 20S proteasome for survived patients was 4,910 ng/ml while for non-survived patients it was 4,170 ng/ml. Regarding the relation of survival and percentage of apoptotic lymphocytes, the median value of for those who survived was 3.8%, while for non-survived patients it was 3.3%. There was no significant correlation for 20S proteasome and the percentage of apoptotic lymphocytes to the survival rate in both groups separately. A significant positive correlation between the two measured factors 20S proteasome and apoptotic total lymphocytes, B lymphocytes and T lymphocytes in both the sepsis and non-sepsis groups was detected (Tables 7 to 9, and Figures 6 to 11).

**Table 7:** Correlation of survival and 20 S proteasome in the sepsis and non-sepsis groups

20 S proteasome	Alive			Died			Mann-Whitney test	
	Range	Median	Mean rank	Range	Median	Mean rank	*Z*	*P *value
Sepsis group	14,200.00 - 38,450.00	26,150.00	17.69	13,700.00 - 38,200.00	19200.00	14.23	0.992	0.327
Non-sepsis group	1,170.00 - 21,710.00	4,910.00	18.64	12,20.00 - 13,590.00	4170.00	16.92	0.478	0.649

**Table 8:** Correlation of survival and percentage of total apoptotic lymphocytes in the sepsis and non-sepsis groups

Percentage of apoptotic lymphocytes	Alive			Died			Mann-Whitney test	
	Range	Median	Mean rank	Range	Median	Mean rank	*Z*	*P *value
Sepsis group	9.40 to 18.40	12.20	18.12	8.20 to 15.90	10.50	13.41	1.350	0.180
Nonsepsis group	1.50 to 9.60	3.80	18.80	1.80 to 7.40	3.30	16.65	0.598	0.555

**Table 9:** Correlation between 20 S proteasome and percentage of apoptotic lymphocytes in the studied groups

		*R *value	*P *value
Sepsis group	Apoptotic (total)	0.746	0.000*
	Apoptotic (B lymphocytes)	0.642	0.000*
	Apoptotic (T lymphocytes)	0.636	0.000*
Nonsepsis group	Apoptotic (total)	0.768	0.000*
	Apoptotic (B lymphocytes)	0.636	0.000*
	Apoptotic (T lymphocytes)	0.766	0.000*

*Significant change, *P *< 0.05.

## Discussion

Critically ill patients, whether suffering from sepsis or not are classified as high-risk patients as regard morbidity and mortality. Previous studies have become growing up to evaluate the immunological state in different diseases, in addition different immunologic markers were previously measured separately to evaluate these patients. In the present study, we tried to evaluate two immunological factors as diagnostic and prognostic markers separately and as both of them together to determine their correlation.

The present study monitored serum levels of 20S proteasome, it was increased in both septic and non-septic critically ill patients compared to healthy controls, with a statistically significant increase in septic patients compared with non-septic patients. The median level in the septic group was 11-fold higher than the control group, while in the non-septic group it was 2 fold higher than in the control group. In their studies, Roth and colleagues and Dutaud and colleagues demonstrated elevated serum 20S proteasome in both septic and control groups, which was 2,157 ± 273 ng/ml and 2,319 ± 237 ng/ml, respectively [12,13]. Other studies revealed that elevated 20S proteasome usually occurs in variable conditions demonstrating cellular damage and catabolic activity, such as sepsis, trauma and muscle proteolysis, this is explained by the rapidity of the cellular degradation occurring in these conditions [14]. Elevated 20S proteasome was also noticed in variable autoimmune disorders such as systemic lupus and rheumatoid arthritis, and this elevation was closely related to the periods of disease activity. [15]

The activation of both B lymphocytes and T lymphocytes occurring in sepsis usually leads to formation of aberrantly reactive B lymphocytes and T lymphocytes causing an immunodeficient state in those septic patients [16,17]. Hotchkiss et al., [1] and Bourboulis et al., [18] demonstrated a statistically significant increase in the percentage of dead lymphocytes in septic patients infected with Gram-negative bacteria in comparison to the control group. Baize et al., [19] demonstrated similar increase in the percentage of dead lymphocytes in patients suffering from sepsis.

The present study revealed that either 20S proteasome or the percentage of dead lymphocytes had not any significant correlation separately to the prognosis in both septic and non-septic critically ill patients, while correlation of both 20S proteasome or the percentage of dead lymphocytes was found to have moderate positive correlation in both sepsis and non-sepsis groups.

Previous studies reported increased lymphocyte death (apoptosis) as an evident finding in critically ill septic patients, which was related to the status of humoral immunity and the prognosis of these patients [14,20]. The present study demonstrated that both 20S proteasome and the percentage of lymphocyte death values in critically ill patients could be a good predictor for the prognosis in these patients. This study thus hypothesized that the combined monitoring of both 20S proteasome and the percentage of lymphocyte death could be a potent prognostic predictor in critically ill patients.

## Conclusions

Elevated serum 20S proteasome in critically ill patients is related to an increased rate of muscle breakdown during their critical illness. Increased lymphocyte apoptosis is a sensitive marker in severe inflammatory states. The correlation between 20S proteasome and the percentage of apoptotic lymphocyte in critically ill patients could be a good predictor of patient outcome, prognosis and survival.

## Key messages

• Critically ill patients, whether septic or non-septic, usually have reduced humoral immunity.

• Immunologic markers in critically ill septic and non-septic patients are more valuable in predicting prognosis than other biologic markers.

• The 20S proteasome as a part of proteasome complex is elevated in critically ill patients, whether septic or non-septic, and this elevation is partially due to enhanced and increased cellular damage and partly due to reduced immunity and altered immune response.

• Increased lymphocyte death is another immunologic prognostic marker occurring in critically ill patients, both septic and non-septic.

• Joining 20S proteasome and increased lymphocyte death together could have a more prognostic value in predicting survival in these patients than each of them when measured separately.

## Abbreviations

7-AAD: 7-aminoactinomycin D; ELISA: enzyme-linked immunosorbent assay; FITC: fluorescein isothiocyanate; ICU: intensive care unit; PBS: phosphate-buffered saline; SOFA: Sequential Organ Failure Assessment.

## Competing interests

The authors declare that they have no competing interests.

## Authors' contributions

AAY prepared the manuscript, and followed up the patients. GAS participated in the design of the study, prepared the laboratory results and wrote the related parts. MMM participated in the laboratory results, writing the related parts and interpretation of the results. All authors read and approved the final manuscript.
